# Plant Physiological Analysis to Overcome Limitations to Plant Phenotyping

**DOI:** 10.3390/plants12234015

**Published:** 2023-11-29

**Authors:** Matthew Haworth, Giovanni Marino, Giulia Atzori, Andre Fabbri, Andre Daccache, Dilek Killi, Andrea Carli, Vincenzo Montesano, Adriano Conte, Raffaella Balestrini, Mauro Centritto

**Affiliations:** 1 National Research Council of Italy-Institute of Sustainable Plant Protection (CNR-IPSP), 50019 Firenze, Italy; giovanni.marino@ipsp.cnr.it (G.M.); giulia.atzori@ipsp.cnr.it (G.A.); andre.fabbri@ipsp.cnr.it (A.F.); adaccache@ucdavis.edu (A.D.); andrea.carli@ipsp.cnr.it (A.C.); vincenzo.montesano@ipsp.cnr.it (V.M.); adriano.conte@ipsp.cnr.it (A.C.); raffaella.balestrini@ipsp.cnr.it (R.B.); mauro.centritto@cnr.it (M.C.); 2 Department of Biological and Agricultural Engineering, University of California, Davis, CA 95616, USA; 3 Plant Production and Technologies Department, Konya Food and Agriculture University, 42080 Konya, Turkey; dilek.killi@gmail.com

**Keywords:** photosynthesis, climate resilience, LiDAR, spectral reflectance, hyperspectral, deep-learning, partial least squares regression, phenomics, plant ecophysiology

## Abstract

Plant physiological status is the interaction between the plant genome and the prevailing growth conditions. Accurate characterization of plant physiology is, therefore, fundamental to effective plant phenotyping studies; particularly those focused on identifying traits associated with improved yield, lower input requirements, and climate resilience. Here, we outline the approaches used to assess plant physiology and how these techniques of direct empirical observations of processes such as photosynthetic CO_2_ assimilation, stomatal conductance, photosystem II electron transport, or the effectiveness of protective energy dissipation mechanisms are unsuited to high-throughput phenotyping applications. Novel optical sensors, remote/proximal sensing (multi- and hyperspectral reflectance, infrared thermography, sun-induced fluorescence), LiDAR, and automated analyses of below-ground development offer the possibility to infer plant physiological status and growth. However, there are limitations to such ‘indirect’ approaches to gauging plant physiology. These methodologies that are appropriate for the rapid high temporal screening of a number of crop varieties over a wide spatial scale do still require ‘calibration’ or ‘validation’ with direct empirical measurement of plant physiological status. The use of deep-learning and artificial intelligence approaches may enable the effective synthesis of large multivariate datasets to more accurately quantify physiological characters rapidly in high numbers of replicate plants. Advances in automated data collection and subsequent data processing represent an opportunity for plant phenotyping efforts to fully integrate fundamental physiological data into vital efforts to ensure food and agro-economic sustainability.

## 1. Introduction

Plant physiology is central to photosynthetic performance, growth, and resilience to abiotic stress [1,2]. Physiology represents the functional link between the environment and plant genome [3,4]. Characterization of plant physiology is fundamental to the utility of genome-wide association studies [5,6,7,8]. Analysis of plant physiological status is, therefore, an essential component of plant phenotyping, where the characteristics of a specific genotype are quantified under designated environmental conditions [9,10]. Traditional approaches to plant physiological analyses are often time-consuming and orientated towards a leaf- or organ-level measurement, precluding rapid screening and measurements over a wider canopy level suited to field-based phenotyping. However, given the importance of plant phenomics and precision digital agriculture to future food and bioenergy security [11,12], a number of advances in phenotyping approaches and technologies may reduce this ‘physiological bottleneck’ and facilitate wide-scale rapid analysis of plant physiological status [13,14]. The capability to infer physiological status over wide spatial scales at high temporal resolution is crucial for digital agriculture applications to optimize yield outputs and minimize resource inputs [15]. The aim of this review article is to provide an introduction to traditional plant physiological analyses and their importance in understanding plant performance and stress responses. We then describe phenomic approaches to rapidly gauge these physiological characteristics in relation to high-throughput plant phenotyping.

Concerns regarding the pressures imposed by climate change, population growth, and the loss of productive agricultural land area has led to an expansion in plant phenomic research in recent years. However, the development of plant phenotyping has been impaired by a number of constraints at different scales: the ability to automate large numbers of morphological measurements at high temporal resolution, processing of large volumes of morphological/genetic data, and the capacity to perform detailed physiological analyses. Plant phenotyping efforts are focused towards the identification and development of more productive and climate resilient crop genotypes. Crop productivity and climate resilience are largely determined by the potential to assimilate CO_2_ via photosynthesis, capability to counteract photo-oxidative stress, and the efficient uptake/use of water. Quantification of the photosynthetic, oxidative, and water status of plants requires detailed and time-consuming measurements by highly trained personnel. While it may not be possible to measure these attributes directly, many phenotyping approaches can indirectly infer or estimate these parameters rapidly. After outlining the traditional approaches to quantifying plant ecophysiology, we then describe the theoretical basis and application of rapid screening phenomic approaches that gauge plant ecophysiological status.

## 2. Leaf Gas Exchange

Leaf gas exchange is a core component of plant physiological research [16,17]. The exchange of gases between a part of the leaf, the whole leaf, or the entire plant with the atmosphere underpins photosynthetic CO_2_ uptake and transpiration [18,19]. The expansion in the availability of commercial plant photosynthetic gas exchange systems has led to their increased use in quantifying rates of photosynthesis, biochemical/diffusive limits to CO_2_ uptake, and stomatal physiological behavior in regulating photosynthesis [20,21]. However, these plant photosynthesis systems are relatively complex and prone to mechanical or user error, e.g., [22]. This has impaired their utilization in wide-scale phenotyping studies.

Plant photosynthetic leaf gas exchange systems utilize infra-red gas analyzers to quantify fluxes of CO_2_ and water vapour. A leaf, or a portion of a leaf, is placed in an enclosed leaf cuvette where the concentrations of [CO_2_] and [H_2_O] in the air entering and then exiting the cuvette after passing over the leaf are measured. The delta values of [CO_2_] and [H_2_O] are then used to calculate parameters such as photosynthesis (*P*_N_), stomatal conductance (*G*_s_), and the internal sub-stomatal concentration of CO_2_ within the leaf (*C*_i_), as shown in [23] and the citations contained within. The external concentration of [CO_2_] outside the leaf (*C*_a_), photosynthetic photon flux density (PPFD), temperature, relative humidity, leaf-to-air vapour pressure deficit, and velocity of air flow can be controlled within the leaf cuvette. This enables rapid manipulation of cuvette conditions to quantify responses to specific factors influencing photosynthesis and the exchange of gases between the leaf and the external atmosphere. Many plant photosynthesis systems also include the capacity to simultaneously measure chlorophyll fluorescence (ChlF) parameters alongside leaf gas exchange. 

### 2.1. Instantaneous Point Measurements

Instantaneous point measurements give a ‘snap shot’ of leaf photosynthetic status. Point measurements are the simplest and most rapid type of leaf gas exchange measurement to perform, and are, therefore, the most widely used in plant phenotyping studies. Despite the relative simplicity of instantaneous point measurements, these measurements can be time-consuming. If set controlled conditions of PPFD, temperature, *C*_a,_ and leaf-to-air vapour pressure deficit are utilized within the leaf cuvette, each leaf requires a period of time to adjust to those cuvette conditions, precluding the ability to take large numbers of measurements at the same time/conditions in phenotyping trials of multiple genotypes. Less time-consuming instantaneous point measurements of leaf gas exchange parameters can be performed without using controlled cuvette conditions. Such measurements where PPFD and temperature track ‘ambient’ conditions do not require the same adjustment period for the leaf, but variations in ambient conditions, diminishes comparability between measurements, and may render any phenotypic or treatment effect indiscernible [24,25]. A significant disadvantage of these instantaneous point measurements of leaf gas exchange is that they only reflect photosynthetic status at a single point in time over a comparatively small area of leaf (~1.75 to 6 cm^2^). Moreover, leaf gas exchange measurements tend to be comparatively variable between individual plants (and sometimes between leaves of the same plant). A more comprehensive insight into leaf physiological processes that are relevant to phenotyping can be observed in the more detailed leaf gas exchange measurements outlined below; however, these measurements are more complex and time-consuming than instantaneous measurements, further reducing their applicability for high-throughput phenotyping.

### 2.2. Biochemical Efficiency of Photosynthesis

The rate of photosynthesis is determined by biochemical and diffusive constraints on the uptake and assimilation of CO_2_ [23,26]. The biochemical efficiency of photosynthesis is a key parameter in determining plant growth rate and crop yield [27,28,29,30,31,32,33], and, therefore, of primary importance to plant phenotyping applications. Leaf level rates of photosynthesis in many staple crops such as rice are relatively low [34]. Identification of genotypes with higher leaf level photosynthetic capacities has the potential to positively affect future crop yields [30,35,36], and identification of genotypes that retain biochemical assimilation during abiotic stress such as water deficit or drought can contribute to more climate-resilient agriculture [27,37]. However, analysis of the biochemical efficiency of leaf level photosynthesis is highly time-consuming, and it is not possible to rapidly and accurately assess large volumes of genotypes at sufficient levels of replication [38,39]. 

**Figure 1:**
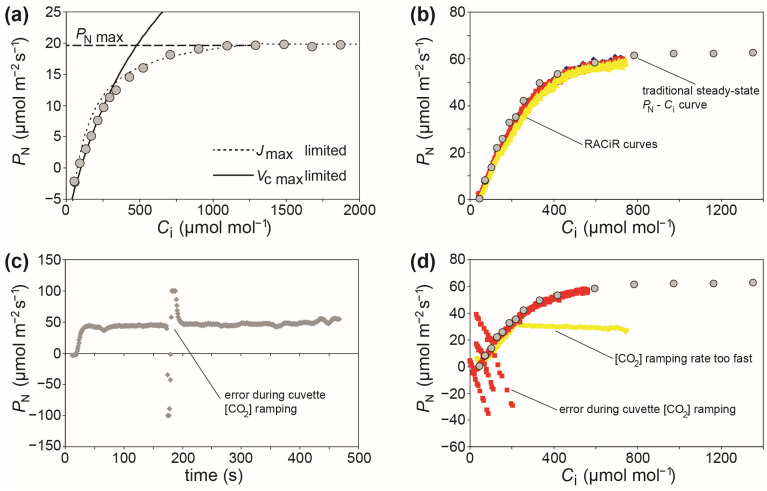
(**a**) *P*_N_–*C*_i_ response curve (*Olea europaea* L.) showing the stage of the curve limited by the rate of electron transport required for ribulose-1,5-bisphosphate (RuBP) regeneration (*J*_max_), the part of the curve limited by the carboxylation capacity of ribulose-1,5-bisphosphate carboxylase/oxygenase (RubisCO) (*V*c_max_), and the maximum rate of photosynthesis at *PAR*_sat_ and high [CO_2_] (*P*_Nmax_), grey circles indicate steady state measurements of photosynthetic gas exchange taken at each [CO_2_] level; (**b**) RACiR curves (red, yellow, and blue symbols) overlain with a traditional *P*_N_–*C*_i_ steady state response curve (*Phragmites australis* (Cav.) Trin. ex Steud.); (**c**) example of an error during [CO_2_] ramping that can affect post-processing of a RACiR curve, and; (**d**) the results of the [CO_2_] ramping error outlined in (**c**) on the corrected RACiR curve (red symbols) of a *P. australis* leaf, alongside a RACiR error caused by the use of an excessively fast [CO_2_] ramping rate (yellow symbols).

The most commonly used method to gauge plant photosynthetic capacity in vivo involves the use of photosynthetic leaf gas exchange systems to perform photosynthetic response curves, where the *P*_N_ is measured relative to increasing steps in the concentration of [CO_2_] (commonly known as *A*–*C*_i_ curves where, instead of *P*_N_, *A* stands for the assimilation rate of CO_2_) (Figure 1).

Figure 1 shows a typical *P*_N_–*C*_i_ curve characteristic of a C3 plant Michaelis–Menten enzymatic reaction. Photosynthesis is limited by the availability of CO_2_ at the initial part of the curve, and this represents the maximum rate of carboxylation of ribulose-1,5-bisphosphate carboxylase/oxygenase (RubisCO) (*V*c_max_). At higher levels of [CO_2_], *P*_N_ is no longer limited by substrate availability, but the regeneration of ribulose-1,5-bisphosphate (RuBP) (*J*_max_). The parameters *V*c_max_ and *J*_max_ are critical components of crop and climate models [19,40,41], and would likely be integrated into digital agriculture applications [42,43]; thus, demonstrating their importance to phenotyping of plant physiology [30]. More detailed reviews of *P*_N_–*C*_i_ curves can be found in Sharkey et al. [21], Centritto et al. [44], Ethier and Livingston [45], and Duursma [20]. The parameters derived from these *P*_N_–*C*_i_ curves are fundamental to the characterization of the biochemical efficiency of *P*_N_, but these measurements are time-consuming (each curve taking ~40–120 min), prone to error, and require expensive equipment operated by staff trained in leaf gas exchange. This makes traditional steady state photosynthetic response curves unsuited to high-throughput phenotyping systems [38,39,46].

Given the importance of the biochemical efficiency of photosynthetic CO_2_ assimilation to crop productivity [24,30], attempts have been made to adapt these measures for use in phenomic applications. Newer plant photosynthetic gas exchange systems are able to continuously ‘ramp’ [CO_2_] in the leaf cuvette and dynamically measure the concurrent effect on *P*_N_. These response curves are known as RACiR curves, standing for “Rapid *A*-*C*_i_ Response” [46]. The RACiR curves can be performed in ~5–15 min, significantly increasing the capability to perform detailed physiological analysis of photosynthetic capacity [31,46,47,48,49]. However, a number of limitations constrain the potential widespread application of RACiR curves: (1) RACiR curve measurements require set-up time to perform empty cuvette measurements to determine the lag in the system between the reference and sample IRGAs [46]; (2) RACiR curves require a degree of prior knowledge of the plant species under analysis (for example, if there is a wide range of *G*_s_ values of plant varieties in a phenotyping study, this may require adjustment of the [CO*_2_*] ramping speed between varieties) [50,51]; (3) preparation of the RACiR curves involves more extensive processing of data after measurements than traditional *P*_N_–*C*_i_ curves, and this reduces the opportunity to identify and correct any errors during measurements (Figure 1b), and; (4) variations associated with photosystem I electron transport dynamics [52]. To minimize some of these limitations by eliminating data post-processing, a recent modification of the method known as the single-step CO_2_ response (SSCO_2_R) has been proposed. This involves equalizing the pathways (or air volume) travelled by the reference and analysis sample air flows to the respective IRGAs during the CO_2_ ramping process [53].

The parameters *V*c_max_ and *J*_max_ are highly significant to models of photosynthesis [26,54], stomatal behavior [55,56,57], crop yield [58,59], and climate modelling [19], but the application of *V*c_max_ and *J*_max_ is constrained by the length of time and the complexities outlined above in performing sufficient numbers of reliable *P*_N_–*C*_i_ curves. To speed up data collection, *V*c_max_ has also been calculated from single-point measurements of light-saturated photosynthesis (*P*_N sat_) [60,61]. This ‘one-point method’ assumes that in the light-saturated state, the leaves are limited by CO_2_ availability, allowing calculation of *V*c_max_ from the C3 photosynthesis model [26,62]. Accurate prediction of *V*c_max_ from a single-point measurement of leaf gas exchange requires the *P*_N_ of the leaf to be limited by CO_2_ availability, knowledge/estimation of respiration in the light, and for the leaf to be fully acclimated to saturating PPFD. In cases where these conditions are not met, estimates of *V*c_max_ may be rendered inaccurate [63,64]. This uncertainty leaves the more time-consuming *P*_N_–*C*_i_ curves as the benchmark methodology for the determination of *V*c_max_ and *J*_max_.

### 2.3. Light-Use Efficiency and Photoprotection

The conversion of light energy to sugars underpins plant growth, and is central to crop yield [65]. However, excess light can induce photo-oxidative stress through the production of reactive oxygen species [66]. During episodes of abiotic stress, such as drought, when the capacity to utilize energy for photochemistry declines, a greater proportion of the absorbed light energy must be dissipated as heat or re-emitted at a longer, less energetic wavelength as ChlF [67,68]. It is, therefore, important for crop phenotyping efforts to enhance productivity and climate resilience to characterize the physiology of light harvesting and photoprotection.

Photosynthetic light capture and use can be analyzed a number of ways using leaf gas exchange, often in combination with ChlF [69]. The most common approach is to measure *P*_N_ at decreasing/increasing steps of PPFD using the LED lights within the plant photosynthesis system leaf cuvette. This enables calculation of the maximum quantum efficiency ($\Phi_{{CO}_{2}}$), the light saturation point (PPFD above which *P*_N_ no longer increases: *P*_N sat_), identification of levels of PPFD where photoinhibition may occur [38,70], and respiration in both the light (*R*_light_) and the dark (*R*_dark_) [71]. These physiological parameters are useful in identifying crop varieties with greater quantum efficiencies that are more likely to correlate to yield [72].

Light response curves incur many of the same impediments for high-throughput phenotyping as *P*_N_–*C*_i_ curves. Despite their utility in the accurate characterization of light harvesting and protective physiologies, light response curves using leaf gas exchange are likely to be unsuitable for phenotyping in comparison to the leaf-level sensor and remote-sensing-based approaches outlined below. Nevertheless, light response curves derived from leaf gas exchange may still be required to ‘support’ data and observations derived from more rapid approaches suited to high-throughput phenotyping.

#### Photosynthetic Response to Variable Growth Conditions

Plant responses to variations in radiation are becoming increasingly important to efforts to improve crop productivity [65,73,74]. Under normal growing conditions in the field, the light environment experienced by leaves can be highly variable (passing clouds, temporary shading from other leaves in the canopy, changes in leaf orientation as the canopy moves due to wind, etc.) Small increases in the efficiency of plants to use this heterogeneous light would translate into improved yields when scaled over whole canopies and an entire growing season [74,75]. The mechanisms involved in the transitions between photochemical and protective energy dissipation may be evident in canopy-level measurement of sun-induced fluorescence (see Section 3.1) [76]. This improved efficiency in photosynthetic light harvesting is related to the physiological processes involved in the transitions between light capture and energy dissipation (outlined in Section 3—ChlF), and also physiological stomatal behavior through stomatal control of *P*_N_ [77,78].

Stomatal physiological regulation plays a central role in plant carbon and water efficiencies [79,80]. Analysis of *G*_s_ values over time to changes in factors such as PPFD, [CO_2_], chemical signals of drought, or leaf-to air vapour pressure deficit enables quantification of stomatal physiological responsiveness [81,82,83,84]. These ‘stomatal kinetics’ are performed by placing a leaf within a cuvette and monitoring *G*_s_ over time while cuvette conditions are adjusted [38]. Stomatal kinetic responses can be used to show physiological differences between varieties that may translate into enhanced productivity [85,86], resilience to stresses such as drought [83,87], or fumigation with atmospheric pollutants [88]. However, measurement of stomatal kinetics using leaf gas exchange is particularly time-consuming given the requirement for the leaf to completely adjust to cuvette conditions prior to recording the full extent of any response to a change in cuvette conditions and the inherent variability in many *G*_s_ measurements requiring high numbers of replicates for statistical robustness [38].

## 3. Chlorophyll Fluorescence (ChlF)

The majority of plant photosynthesis systems offer the opportunity to simultaneously measure ChlF alongside leaf gas exchange parameters by integrating pulse amplitude modulation (PAM) fluorometers within the leaf cuvette. While the ChlF measurements used in PAM fluorometer systems may differ somewhat to those used in high-throughput phenotyping and precision agriculture, the underlying principles and many of the parameters recorded are the same. Detailed reviews of ChlF applications in plant physiology and phenotyping can be found in Maxwell and Johnson [89], Strasser et al. [90], and Kalaji et al. [91]. Analysis of ChlF can provide insights into plant physiological processes, and given the nature of the measurement of ChlF, it may be more suited to high-throughput phenotyping applications than leaf gas exchange. Chlorophyll fluorescence measurements are based on the principle that changes in the amounts and proportions of radiation absorbed by chlorophyll for photochemistry, dissipated as heat, or re-emitted as ChlF can provide an indication of the physiological status of the plant [92]. The amount of absorbed radiation emitted as ChlF in the wavelength bands 685–690 nm (red) and 720–735 nm (far-red) ranges from 0 to 10% depending upon the status of photosynthesis [93]. Fluorescence in the far-red wavelengths (*F*_far-red_) is mainly associated with fluorescence from photosystem I (PSI). Fluorescence derived from PSII mainly occurs in the red band (*F*_red_), and is more sensitive to short-term variations in photosynthetic performance than *F*_far-red_ due to the availability of PSII reaction centres associated with photochemical and non-photochemical quenching [92,94,95]. The constraint to the widespread use of ChlF techniques in phenotyping and digital agriculture is the comparability, quantification, and standardization of different indices dependent upon the type/optical leaf properties of the plant and light environment used to stimulate ChlF [96]. Measurements of ChlF are frequently categorized as active (where a pulse of light is applied to the leaf) or passive (where ChlF is monitored under normal light conditions) [97]. An impediment to the use of ChlF for phenotyping at high temporal frequency over a large spatial area is the accurate determination of fluorescence due to it constituting a small proportion of total reflectance in both of the fluorescence wavelength bands, and confounding factors such as scattering of *F*_red_ re-absorption [97,98,99].

**Figure 2:**
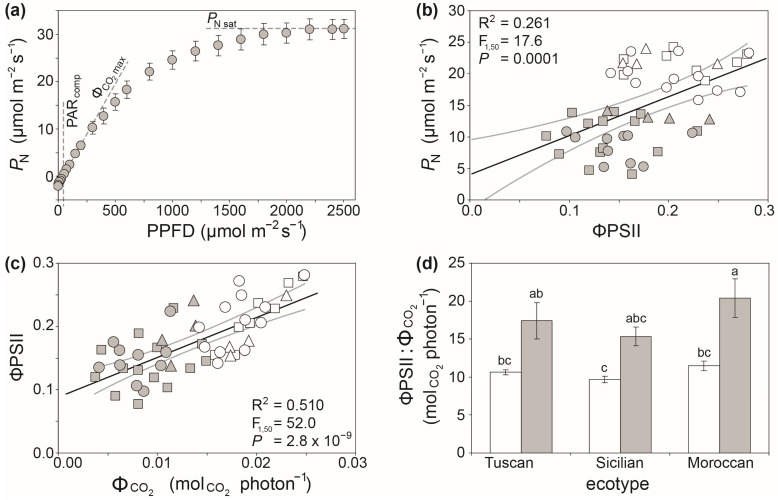
(**a**) The light compensation point (PAR_comp_), maximum quantum efficiency of CO_2_ assimilation ($\Phi_{{CO}_{2max}}$), and light-saturated rate of photosynthesis (*P*_N sat_) from a light response curve of wheat (*Triticum aestivum* L.); (**b**) relationship between *P*_N_ measured using gas exchange and ΦPSII measured using ChlF of well-watered (white fill) and drought-stressed (grey fill) Moroccan (circle symbol), Sicilian (triangle symbol), and Tuscan (square symbol) ecotypes of *Arundo donax*, black central line indicates the regression best-fit, the two grey lines either side indicate 95% confidence intervals of the mean; (**c**) relationship between the actual quantum efficiency of PSII in the light-adapted state (ΦPSII) determined using ChlF and quantum efficiency determined using leaf gas exchange ($\Phi_{{CO}_{2}}$) of *A. donax* (symbols and statistical analysis as in (**b**), and; (**d**) the ratio of ΦPSII to $\Phi_{{CO}_{2}}$ of the *A. donax* genotypes under well-watered (white fill) and drought-stressed (grey filled) conditions consistent with an increase in the proportion of energy utilized via photorespiration in the drought-stressed plants, letters indicate homogeneous groupings indicated by a one-way ANOVA with an LSD *post-hoc* test. Recalculated from Haworth et al. [109] and Riggi et al. [72].

The majority of ChlF measurements used in plant physiology utilize measurement of *F*_red_ to assess the performance of PSII due to its greater sensitivity to growth conditions, [90,92]. Nevertheless, dual-PAM systems allow analysis of fluorescence associated with both PSI and PSII [100,101]. One of the most widely used active ChlF parameters in plant physiology is the actual quantum efficiency of photosystem II in light-adapted conditions (ΦPSII) [102]:

$$\Phi PSII=\frac{{F_{m}}^{\prime }-F_{s}}{{F_{m}}^{\prime }}$$

where the steady state fluorescence (*F*_s_) is recorded prior to a high-intensity saturating pulse of light being applied to the leaf (or a series of sub-saturating multi-phase pulses: [103]) to determine the maximum fluorescence under steady-state conditions in the light (*F*_m_’). The ΦPSII parameter is more sensitive to abiotic stress than the maximum quantum efficiency of PSII (*F*_v_/*F*_m_: where *F*_v_ indicates the maximum variable fluorescence, and *F*_m_ the maximum fluorescence after a saturating pulse of light of a dark-adapted leaf where all PSII reaction centres are available) [104] that requires dark adaptation to fully open the PSII reaction centres [90]. Moreover, ΦPSII often correlates closely with the rate of *P*_N_ measured using leaf gas exchange (Figure 2b). The ΦPSII determined from ChlF gives an indication of photochemistry, specifically both *P*_N_ and photorespiration (*R*_PR_) [102,105]. In contrast, the $\Phi_{{CO}_{2}}$ determined from gas exchange quantifies the amount of CO_2_ assimilated through *P*_N_ relative to the incident PPFD [70]. As plants become more drought-stressed, an increasing proportion of energy utilized for photochemistry will drive the oxygenation of RubisCO, as *R*_PR_ serves as a sink for excess electrons [106,107]. An increase in ‘wasteful’ photorespiration due to the loss of previously fixed carbon [108] may be indicated by a shift in the ratio of ΦPSII to $\Phi_{{CO}_{2}}$ [38]. In this context, the comparison of quantum efficiencies derived from ChlF and leaf gas exchange may be useful in identifying crop species and varieties with enhanced tolerance to abiotic stress such as drought (Figure 2d) [109]. However, under a strong prolonged drought stress, as photochemistry declines and non-photochemical energy dissipation increases [110], the utility of comparison of ΦPSII and $\Phi_{{CO}_{2}}$ will become compromised. 

A new generation of comparatively low-cost handheld optical sensors (such as the PhotosynQ MultispeQ, PhotosynQ Inc., East Lansing, MI, USA) can enable the rapid collection of large volumes of GPS linked leaf-level ΦPSII. The use of sequential pulses of light also allows the PhotosynQ to extrapolate *F*_m_’ to determine NPQ [111]. Moreover, porometer models such as the LiCor Li-600 porometer–fluorometer (LI-COR Biosciences, Lincoln, NE, USA) also include IRGAs capable of rapid measurement of water vapour, enabling measurement of *G*_s_ alongside ΦPSII (and other light-adapted ChlF parameters) for fast phenotyping of large numbers of genotypes at sufficient levels of replication. Chlorophyll fluorescence can also be assessed at wider scales over the canopy-level using passive approaches; these are detailed in Section 3.1.

Under constant stable illumination in PPFD (a ‘steady-state’), the average emission of fluorescence is known as ‘steady state fluorescence’ (*F*_s_). An advantage of *F*_s_ as a ChlF parameter is that it can be measured passively, and does not require ‘active’ stimulation with pulses of light, facilitating its use to continuously monitor large numbers of plants [112]. Moreover, *F*_s_ is affected by water [113,114] and heat stress [115]. Steady-state fluorescence can be measured using a handheld fluorimeter or monitored remotely using a fluorimeter or hyperspectral sensor. Moreover, integration of *F*_s_ into ChlF indices utilized as part of sun-induced fluorescence has direct applications in linking leaf-level physiological processes to more rapid wider-scale phenotyping and digital agriculture applications [116,117].

### 3.1. Sun-Induced Chlorophyll Fluorescence 

Sun-induced chlorophyll fluorescence (SiF) uses leaf reflectance spectroscopy to passively measure fluorescence under natural illumination at a range of scales from the leaf to the canopy. As SiF is associated with plant photosynthetic performance, this can allow the rapid screening of large numbers of plants under representative natural growth conditions [99,117,118]. To measure the two wavelength peaks associated with ChlF, it is necessary to quantify the amount of light derived from fluorescence and reflectance. This can be achieved due to the similarity in ChlF wavelengths to absorption bands of oxygen at 687 and 760 nm [119,120]. One difficulty in accurately quantifying SiF is that it accounts for a comparatively low percentage of total reflected radiation [98], and is affected by the amount of oxygen (atmospheric pressure, temperature, and height of spectroscopy measurement) [121], scattering by aerosols [122], canopy structure [123,124], accurate measurement of the quantity/spectra of incoming radiation [94,125], and the reabsorption for *P*_N_ of light emitted in the *F*_red_ wavelengths [99,126].

Sun-induced fluorescence can be gauged quantitatively as amounts of radiation or as relative indices, e.g., [95,123]. The use of indices may enable greater comparison between measurements taken at different time-periods under different light conditions (this is outlined in more detail in Section 6). The emission of *F*_far-red_ has been used to gauge gross primary productivity [118,127] due to its relationship with PSI [92]. Photosystem II fluorescence in the 687 nm red wavelength may provide more information on photosynthetic performance over the short-term, but is subject to greater uncertainties associated with re-capture of fluorescence radiation [126]. This has led to the use of the ratio of red to far-red fluorescence (*F*_ratio_) [99] that provides an indication of the respective fluorescence yields of PSI and PSII [128]. Heat stress, water deficit, soil nitrogen deficiency, canopy structure, and foliar chlorophyll content all affect *F*_ratio_ [117]. Sun-induced fluorescence can also be standardized relative to the amount of incoming radiation to reduce variation between measurements taken at different times and under different conditions. The fluorescence quantum yield (*F*_yield_) is the ratio of the emission of fluorescence to the level of PPFD. The level of fluorescence can also be standardized relative to total vegetation reflectance at the 687 and 760 nm wavelengths [94]. The fluorescence emission of plants may also be affected by the physical properties of the canopy and leaf structure [123,129]. The fluorescence correction vegetation index (FCVI) is the difference between near-infrared and visible reflectance (400–700 nm), and attempts to characterize PAR absorption and SiF scattering processes [124]. The use of FCVI alongside *F*_far-red_ may provide context to the photosynthetic processes associated with fluorescence emission through characterization of light absorption and scattering properties of the canopy [116,124].

Sun-induced fluorescence may also provide valuable information on plant photosynthetic status when used in conjunction with other spectral indices. The photochemical reflectance index (PRI) gives an indication of the performance of the xanthophyll cycle and the dissipation of excess energy as NPQ [130]. Therefore, analysis of SiF and PRI can provide valuable insights into the physiological status of plants, specifically the efficiency of light harvesting and protection from excess light [95,116]. Likewise, comparison of SiF to the widely used normalized difference vegetation index (NDVI) [131], which uses visible and near-infrared reflectance to quantify green biomass [132,133], can be used to infer shifts in PSII function relative to chlorophyll content [99], the absorption of PAR [124], and the vegetated fraction of the surface [134,135]. However, SiF is more variable than NDVI due to the dependence upon the level of PPFD [125,135], potentially impairing the comparability of measurements over temporal and spatial scales. 

Sun-induced fluorescence enables the rapid assessment of photosynthetic activity of large numbers of plants under natural growth conditions. However, uncertainties associated with the passive measurement of fluorescence may reduce the efficacy of SiF as a stand-alone approach to gauging plant physiological status for phenotyping purposes. Nevertheless, the use of absolute and relative indices of SiF alongside other spectral indices or LiDAR analysis of canopy structure may enable the use of SiF as a component of plant phenotyping applications.

## 4. Handheld Optical Sensors

An increase in the availability of handheld optical sensors capable of rapid measurement of a range of plant physiological processes has significant applications for high-throughput phenotyping. Handheld sensors have been utilized widely for analysis of the efficiency of PSII in both dark- and light-adapted states [67,136]. The absorbance spectra of chlorophyll has also been extensively utilized to non-destructively quantify leaf chlorophyll content, with the Konica Minolta SPAD (Konica Minolta Inc., Tokyo, Japan) being the most widely used instrument [137,138].

**Figure 3:**
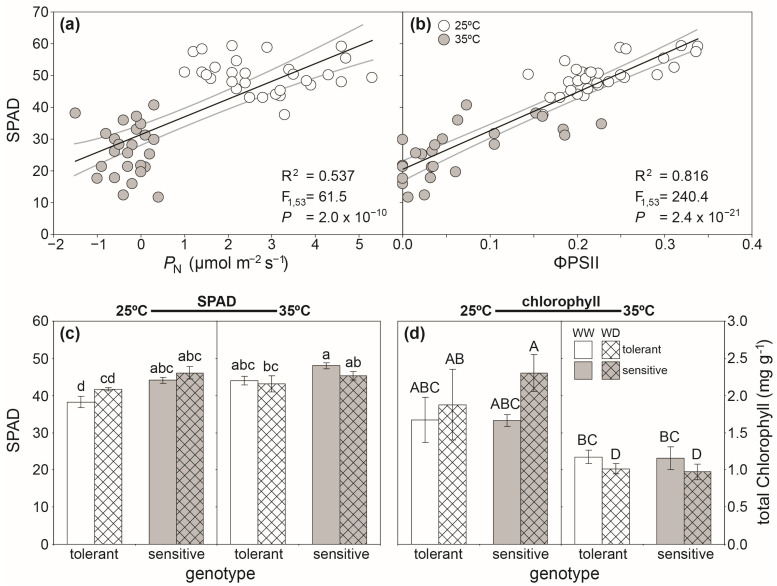
The relationship between SPAD values and leaf gas exchange values of photosynthesis (**a**) and chlorophyll fluorescence analysis of the actual quantum efficiency of photosystem II (ΦPSII) (**b**) of ginkgo (*Ginkgo biloba* L.) grown at 20/25 °C (white fill) and 30/35 °C (grey fill) [146]—the solid black lines indicate linear regression, grey lines either side of linear regression indicate 95% confidence intervals of the regression line. The use of SPAD (**c**) and spectrophotometric quantification of chlorophyll content per dry weight of leaf (**d**) to phenotype the effect of well-watered (WW: open fill) and water deficit (WD: hashed fill) irrigation on drought-tolerant (white fill) and drought-sensitive (grey fill) sunflower (*Helianthus annuus* L.) [67]. Error bars indicate one standard error either side of the mean. Letters indicate significant difference between groups in SPAD (lower case) and chlorophyll content per dry weight of leaf (upper case letters) values using a one-way ANOVA and LSD post hoc test.

A number of phenotyping studies have selected crop varieties on the basis of the foliar concentration of chlorophyll and RubisCO [139,140,141], due to the association between leaf-level *P*_N_ and yield [34]. Foliar chlorophyll concentration often correlates with direct leaf gas exchange of photosynthesis (Figure 3a) and ΦPSII (Figure 3b). Handheld SPAD estimates of leaf chlorophyll content also closely correlate to specific spectral reflectance indices (e.g., NDVI) [142], allowing rapid screening of large numbers of plants for foliar chlorophyll content [143,144,145]. Handheld SPAD measurements can be useful in differentiating phenotypic differences between crop genotypes in their response to factors such as heat and water-deficit stress (Figure 3c). However, changes in leaf thickness associated with specific stresses can influence the effectiveness of SPAD measurements of foliar chlorophyll content on an area basis. As the effect of changes in leaf thickness that may only become evident when chlorophyll is assessed per unit dry mass of leaf tissue (Figure 3d).

The absorbance characteristics of other plant pigments can be used to non-destructively characterize physiological status. The Dualex (Force A, Orsay, France) and Opti-Sciences MPM-100 multi pigment meter (Opti-Sciences Inc., Hudson, New Hampshire, USA) simultaneously quantify the absorbance of chlorophyll, anthocyanin, and flavonols to produce an index for leaf nitrogen levels (nitrogen balance index or nitrogen–flavonol index) [147,148,149]. These indices are useful for rapid phenotyping and digital precision agriculture due to the photo-protective roles of anthocyanins and flavonols, alongside the importance of foliar nitrogen levels to rates of *P*_N_ and growth [150].

The advances in LED technology that have driven the expansion in the availability and applications of handheld plant physiological sensors have also enabled the development of handheld sensors capable of assessing the spectroradiometric properties of leaves. The potential uses of spectroradiometric indices for plant phenotyping to gauge *P*_N_, plant water status, and photo-protection are outlined in more detail in Section 6. Nonetheless, the capability to measure leaf spectra rapidly using comparatively low-cost handheld sensors is highly suited to rapid phenotyping of plant physiological status in comparison to slower, more complex leaf gas exchange measurements.

## 5. Plant Water Status

The efficient uptake and use of water is a key component of crop performance and of central importance to the tolerance of abiotic stresses such as heat, drought, and salinity [2,77,151,152]. Several approaches can be used to gauge plant water content for physiological analysis. Among the most frequently used [153] are weighing lysimeters that determine evaporation from the soil–plant system (ET) through weight-based estimates of ET and drainage [154], a parameter that is directly related to plant water status. Lysimeters are mainly used under controlled or semi-controlled conditions where the level of irrigation can be tightly regulated. When used for phenotyping, lysimeters can be combined with other measuring tools, such as soil and atmospheric probes; nevertheless, the main limitation associated with lysimeters is the need of controlled or semi-controlled environments, constraining their application to rapid phenotyping. Lysimeters are adaptable to open-field conditions when provided with top covers to avoid rainwater infiltration that would prevent accurate measurement of transpiration. Nevertheless, even if adaptable to open field, such lysimeters systems still rely on specific portions of soil being isolated using pots, buckets, or tanks dependent upon the volume of soil and size of plants to be investigated [155].

Other physiological approaches can be used to gauge plant water content for physiological analysis, including stomatal conductance (*G*_s_), leaf turgor, leaf thickness, water potential (Ψ), relative water content (RWC), and sap flow (SF) [156]. Nevertheless, the measurement of such parameters can be destructive and/or time-consuming. This makes these measurements unsuitable, when used in isolation, for wide-scale phenotyping studies or continuous measurement for precision agriculture [157]. Leaf relative water content (RWC) can be calculated by comparing the fresh, turgid, and dry mass of a leaf without the need for specialized equipment [158]; however, alongside being a destructive analysis, this is a time- and labor-consuming approach unsuited to rapid high temporal resolution screening. The use of a Scholander pressure bomb to determine the water potential (Ψ) of different tissues allows quantification of the turgor pressure associated with the osmotic flow of water from the soil to the roots and upwards towards the photosynthetic organs [159,160]. The lower the water potential of the plant relative to the water potential of the soil in the root zone, the greater the capacity of the plant tissue to osmotically draw water [161,162]. Values of leaf and stem water potential not only decline as soil water availability decreases during drought, but also over the course of a day, with the most physiologically informative readings of Ψ occurring at pre-dawn (when plant water potential has equalized with the soil) and midday (the time of maximum transpirative demand) [163,164,165,166]. The cell pressure probe technique used to measure leaf turgor is invasive and unsuitable for long-term outdoor applications [167,168]. Leaf thickness measurements can require cutting the leaf when using micrometers to determine a pressure–volume curve [169]. Nuclear magnetic resonance can also be used to non-destructively gauge leaf water [170,171]. However, nuclear magnetic resonance requires highly trained personnel, expensive equipment, detailed post-processing of data, and an extended period for each measurement, making it unsuited to low-cost, fast phenotyping applications. Measurement of the rate that sap flow ascends a plant can determine its transpiration rate, and can, thus, be used an indicator of plants water status [156]. These measurements can be made using two main approaches: the first calculates the sap flow rate through the heat balance method, which is destructive and unsuited for plant phenotyping., while the second method calculates sap flux density using the heat pulse or continuous thermal dissipation methods [172]. The heat pulse method is described in more detail in Section 5.1 below.

### 5.1. Analysis of Whole Plant Water Relations

Plant water potential can be measured on leaves (Ψ_leaf_), with this value representing local leaf water demand. The water potential of the xylem (Ψ_xylem_) reflects whole plant transpiration with soil and root/soil hydraulic conductivity. Stem water potential (Ψ_stem_) is measured on a non-transpiring leaf, as when leaves do not transpire, their water potential is considered to correspond to stem water potential [156]. Several studies have shown that Ψ_stem_ can be an effective water deficit indicator, and a more representative measure of plant water status than Ψ_leaf_ [173,174,175]. Methods for water potential determination include the Scholander pressure chamber described above, thermocouple psychrometers, and microtensiometers. The first two methods do not measure water potential continuously and are labor- and time-consuming, whereas microtensiometers offer the option to continuously monitor water status [176]. These devices measure water potential based on a microelectromechanical pressure sensor that is embedded in the trunk and directly measures stem water potential [156]. Microtensiometers provide accurate continuous measurements of Ψ_stem_ in trees during the growing season across a wide range of environmental conditions and soil water content values [176]. However, in irrigated grapevine, microtensiometers appeared to be insensitive under high VPD and did not reflect diurnal and seasonal changes in water potential [177].

The sap flow rate in plants is correlated to the rate of transpiration, and can be used as an indicator of plant water status. The two main methods to determine sap flow rate are the heat pulse and the continuous heat methods, highly accurate, and low-cost approaches to determine sap flow in roots, stems, and branches [156]. Various types of sensors exist that measure temperature changes in the xylem following heat application [178]. The transpiration rate is not then directly measured from sap flow, but from the transfer and movement of heat within the xylem [179]. These measurement techniques are consistent with weighing lysimeters. Moreover, the use of a low-cost technology with high time-resolution and automated data collection and storage makes heat pulse and continuous heat approaches valuable tools for plant water status determination. Limitations have been observed in heat pulse method sensors, which, while accurate in correlating heat velocity with rates of transpiration, were poor in quantifying transpiration [179]. In contrast, the thermal dissipation probe used in the continuous heat method was found to be affected by external temperature variations and requires species-specific calibration to allow accurate sap flow measurement. 

### 5.2. Analysis of Canopy-Level Water Relations—Infrared Thermography

**Figure 4:**
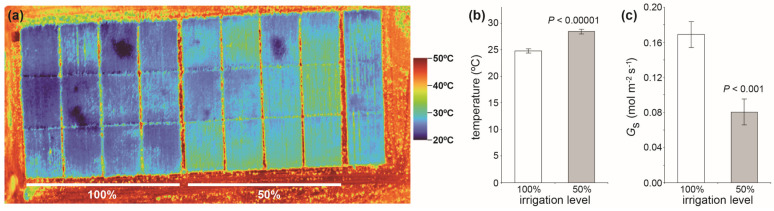
(**a**) UAV-mounted infrared thermography of cannabis (*Cannabis sativa* L.) receiving full (100% white fill) and water deficit (50% grey fill) irrigation. (**b**) Temperature measurement of the plots shown in panel a (one-way ANOVA: F_1,22_ = 35.4; *p* = 5.4 × 10^−6^). (**c**) Stomatal conductance (*G*_s_) of the plants measured using a LiCor Li600 porometer–fluorometer (Li-Cor, Inc., Lincoln, NE, USA) (one-way ANOVA: F_1,22_ = 18.0; *p* = 0.0003).

As a complement to the leaf, stem, and whole plant measurements outlined above, the analysis of canopy-level images can also provide information on the water status of large numbers of plants. The use of infrared thermography to measure canopy temperature can provide insights into the water status of large numbers of plants under field conditions or protected cultivation [180]. Leaf temperature correlates to *G*_s_ due to the cooling effect of transpiration, making infrared thermography a useful tool for irrigation scheduling [181]. Infrared thermography is a relatively low-cost approach to monitor the onset of stomatal closure in large numbers of plants subject to water deficit [182] that could be useful in identifying crop varieties with more sensitive physiological stomatal behavior and signalling (Figure 4). Spectral reflectance techniques outlined below in Section 6 also provide insights into canopy water relations that can be correlated to direct measurement of Ψ_plant_, *G*_s,_ and sap flow.

### 5.3. Leaf-Based Sensors

In light of the fundamental role of plant water relations to *P*_N_, rapid, wide-scale, and high-frequency analysis of plant water status is vital to effective phenotyping of crops in terms of productivity and climate resilience. To overcome the previously described constraints associated with traditional methods, a number of non-invasive leaf- or plant-based sensors and remote sensing approaches have been developed to continuously gauge the water content of vegetation. 

Small interconnected data-logging leaf-based sensors can be used to non-destructively indirectly gauge leaf water levels, either through changes in pressure sensors [183,184], variation in spectral reflectance [185], or the attenuation of a known quantity of light passing through a leaf [186]. Pressure sensors such as the ZIM-probe (ZIM Plant Technology GmbH, Hennigsdorf, Germany) function by applying inward physical pressure to both leaf surfaces (in the case of the ZIM-probe, this is achieved using magnets). The counter resistance to this inward pressure against the leaf is related to plant water status: i.e., the more turgid the leaf, the greater the outward counter pressure acting against the probe [183,184,187]. The effectiveness of these pressure probes has been demonstrated through comparison to classical measurement of leaf water potential and stomatal conductance in plants subject to soil drying [188,189,190,191,192]. However, the area of the leaf in direct contact with the pressure probe does not receive light and can become chlorotic, impairing the effective use of the probes over extended durations in crops with low structural investment in leaves [188].

As outlined in the Section 6, the spectral and reflectance properties of leaves can be used to gauge foliar water content by exploiting developments in LED technologies that enable the constant emission of specific wavelengths of light. Novel leaf-based sensors such as the Leaf Water Meter (LWM, PaStella Factory SRLS, Verona, Italy) utilize the absorption of light through the leaf emitted by two LEDs at wavelengths of 1450 and 890 nm to estimate foliar water and dry matter content, respectively. Calibration of these optical leaf absorbance parameters to traditional leaf water potential measurements through soil drying and re-watering cycles demonstrates the efficacy of the sensors [186]. A low-cost hyperspectral sensor has also been used to monitor leaf water content using reflectance of light in the wavelength range 1550–1950 nm that corresponds to the absorption properties of water [185]. Microwave-based sensors might also offer similar possibilities for non-destructive continuous measurement of the volume of foliar water [193].

## 6. Spectral Reflectance 

**Figure 5:**
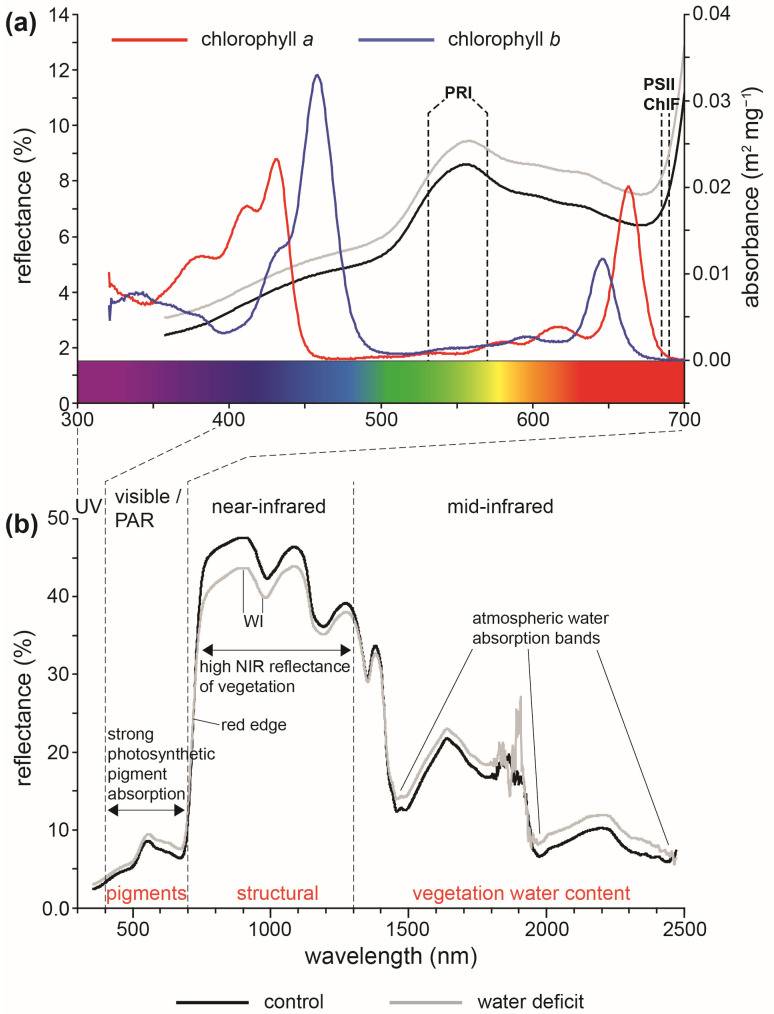
Example of spectral reflectance of an olive leaf under well-watered (black line) and water deficit (grey line) conditions: (**a**) spectral reflectance of the leaf over the wavelengths 300–700 nm that mostly corresponds to visible and photosynthetically active radiation in the 400–700 nm band. Absorption spectra of chlorophyll a and b (data from [201]). Horizontal dashed lines mark the wavelengths utilized for the photochemical reflectance index (PRI—Section 6.1) and PSII chlorophyll fluorescence (Section 3.1). (**b**) Spectral reflectance of the leaf over the wavelengths 300–2500 nm: writing in orange indicates the main factors affecting specific parts of the spectra; key wavelengths and parts of the spectra used to estimate specific parameters such as the water index (WI) or red-edge are marked on the figure [15,196,202,203].

Photosynthesis is powered by the capture of photosynthetically active radiation (PAR) within the 400–700 nm wavelength range for conversion to chemical energy in the form of sugars [194]. Light energy that hits a leaf can either be absorbed, reflected, or transmitted through the leaf (see Section 3 for the fate of absorbed energy) [195,196]. The amounts and proportions of each potential outcome depend upon the intensity and spectrum of the incoming solar radiation alongside the reflective properties of the vegetation [197,198]. The reflective characteristics of a leaf are determined by its biochemical properties and structural attributes [133,199] (Figure 5). To minimize the effect of variation in illumination, the vegetation reflectance is compared to values from a calibrated reflectance panel. The analysis of spectral reflectance usually utilises indices based on wavelengths associated with specific characteristics. These indices are often calculated as the difference between two specific wavelengths or wavelength bands normalized against their sum [200]. Analysis of visible (400–700 nm), near-infrared (700–1300 nm), and mid-infrared (1300–3000 nm) reflectance wavelengths can provide information regarding different aspects of the status of vegetation [196]. Below, we outline the most commonly used spectral indices, their physiological/physical basis, and how these indices could be used to rapidly infer plant physiological, water, and biomass attributes (a more exhaustive list of spectral indices can be found in [200]).

### 6.1. Light Energy Usage and Dissipation

Comparatively little radiation is reflected in the 400–700 visible part of the spectrum that corresponds to the wavelengths used to drive photosynthesis, termed PAR (Figure 5a). Nevertheless, spectral techniques have been widely used to assess the composition of photosynthetic pigments. The concentration of chlorophyll can be estimated from reflectance at ~550 and ~675 nm [204]. Analysis of spectral reflectance in the 680–750 nm wavelength range known as the ‘Red-Edge’ is widely used to gauge canopy chlorophyll content (commonly as the wavelength position of the red-edge, or as the ratio of reflectance at 750 and 700 nm: R_750_/R_700_) and leaf area index [205,206,207]. As outlined earlier in Section 4, the foliar amount of chlorophyll often correlates to leaf-level nitrogen, allowing the use of spectral assessment of chlorophyll content to determine nitrogen levels [208]. Spectral assessment of chlorophyll has also been shown to correlate strongly with handheld optical SPAD estimates [142,209].

The proportion of carotenoids to chlorophyll is indicative of the effect of, and resilience to, photo-oxidative stress [210,211,212]. An increase in the proportion of carotenoids (that preferentially absorb in the shorter more energetic blue wavelength part of the spectrum) relative to chlorophyll *a* (that preferentially absorbs in the less energetic red part of the PAR spectrum) can protect the photosynthetic apparatus through an increase in the capacity of the xanthophyll cycle to dissipate potentially harmful excess energy [213]. This is evident in a shift in the reflectance of wavelengths in the blue (corresponding to the absorption of carotenoids and chlorophyll *b*) and red (corresponding to the absorption of chlorophyll *a*), and can be gauged using spectral reflectance indices such as the normalized difference pigment index (NDPI = [R_680_ − R_430_]/[R_680_ + R_430_]), structural insensitive pigment index (SIPI = [R_800_ − R_445_]/[R_800_ − R_680_]), or simple pigment ratio index (SRPI = R_430_/R_680_) [214,215].

The dissipation of excess light energy as heat via the xanthophyll cycle correlates to reflectance at 531 nm [213]. The photochemical reflectance index (PRI = [R_531_ − R_570_]/[R_531_ + R_570_]) also gauges carotenoid–chlorophyll ratios, and the short-term de-epoxidation state of the xanthophyll cycle [130,202]. This short-term sensitivity to the status of photosynthetic radiation energy harvesting has enabled correlation of PRI with other measures of photosynthetic efficiency such as leaf gas exchange and ChlF (Section 2 and Section 3) [15,157,216]. 

### 6.2. Plant Water Status

As outlined in Section 5 and Section 7, plant water status and the efficiency of water usage/uptake are of central importance to plant physiological processes. A reduction in leaf water content generally induces an increase in leaf spectral reflectance in visible light [217,218] and changes in reflectance wavelengths related to shifts in the proportions of photosynthetic/protective pigments [15,212]. Spectral reflectance shows the greatest sensitivity to foliar water content at wavelength bands corresponding to the absorbance of infrared radiation by water [217]. The water index (WI = R_900_/R_970_) [219,220] and water content reflectance index (WCRI = R_1455_/[R_1272/_R_1455_]) [218] correlate to plant water content. The sensitivity of the WI is also enhanced by normalization against NDVI, as an indicator of plant green biomass [219].

Foliar water content is important in plant responses to salinity and monitoring the vegetation fire risk. The WI is sensitive to salinity stress in barley [221]. A number of normalized difference spectral indices from near-infrared wavelengths were observed to correlate to the leaf moisture content, equivalent water thickness, and relative water content (RWC) of cotton at different salinity levels [222]. This indicates that spectral reflectance may be a valuable tool in monitoring plant response to salt stress when phenotyping varieties capable of cultivation on salt-affected land or irrigated with low-quality saline water.

### 6.3. Biomass and Productivity

Accurate determination of plant biomass is important for monitoring the growth of biomass crops [223] and determination of the optimal timing of harvesting for grain and fruiting crops [224]. Soil and foliage can be differentiated using spectral reflectance through comparison of visible red (R_RED_) and near-infrared (R_NIR_) wavelength bands [132]. The proportion of soil to vegetation cover can also be calculated using visual wavelengths based on the ratio of red to green light with the ratio of blue to green [225]. The normalized difference vegetation index (NDVI = [R_NIR_/R_RED_]/[R_NIR_ + R_RED_]) quantifies the difference between visible and near-infrared reflectance to gauge the amount of green vegetation cover over an area of land [132,133]. The NDVI has been used to assess the proportion of the land surface covered by vegetation [134,135], biomass, nutrient status [226,227,228], vegetation canopy structure [132,229,230], and gross primary productivity [231]. The ‘greenness’ of vegetation expressed by NDVI has also been shown to linearly scale with photosynthetic light capture and rates of CO_2_ assimilation [132,157]. The normalized difference red edge (NDRE) and green vegetation index (GVI) were also used to gauge biomass, but were less effective than NDVI [232,233]. Spectral reflectance may also be utilized alongside LiDAR (Section 7) to produce multi-variate proxy measures of plant biomass and/or canopy architecture [234,235].

### 6.4. Linking Spectral Reflectance to Plant Physiological Status

The spectral reflectance properties of leaves offer an indirect insight into the physiological processes that determine plant productivity and resilience to abiotic stress. As such, spectral indices cannot replace a direct measurement of CO_2_ assimilation or photosynthetic electron transport in terms of an empirical observation. However, as outlined earlier, these direct physiological measurements are highly time-consuming and prone to measurement error. Spectral reflectance measurements can be performed over a wide spatial scale at high temporal resolution, and, thus, are highly suited for phenotyping applications, such as the assessment of large numbers of replicates under field conditions [36,236]. This raises the question of the most suitable approach to gauge plant physiological status from spectral reflectance data.

**Figure 6:**
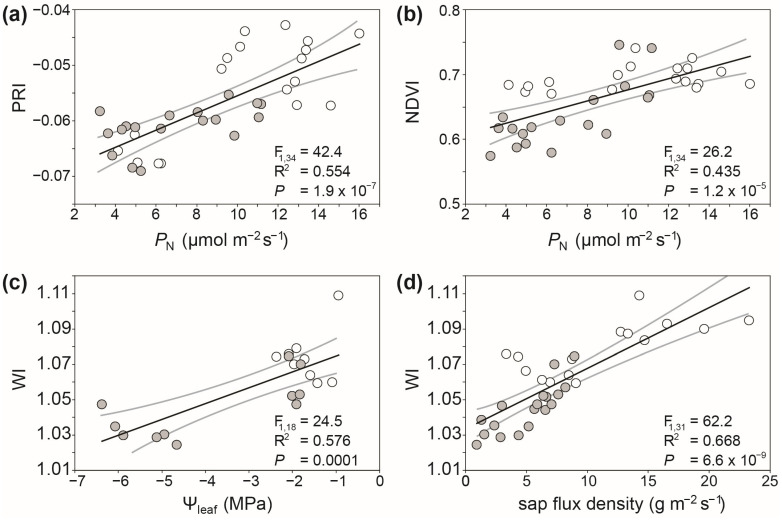
Example of linear regression correlations between spectral reflectance indices and physiological parameters of *Olea europea grown* under full (white fill) and deficit (grey fill) irrigation: (**a**) photosynthesis (*P*_N_) versus photochemical reflectance index (PRI); (**b**) photosynthesis versus normalized difference vegetation index (NDVI); (**c**) leaf water potential (Ψ_leaf_) versus the water index (WI), and; (**d**) sap flux density versus the water index. Linear regression and confidence interval lines as in Figure 3. Re-drawn from Marino, et al. [157].

Linear regression between individual spectral indices and physiological parameters offers a relatively simple method to enable the rapid screening of plant physiological status using spectral reflectance. For example, the spectral reflectance indices of olive trees grown under full and water deficit irrigation correlate with physiological parameters such as photosynthesis, sap flux density, and Ψ_leaf_ (Figure 6) [15,157]. This may enable the use of these calibration training datasets to infer physiological parameters using spectral reflectance. However, these correlations are often spatially and temporally variable depending upon growth conditions [132]. The use of techniques such as partial least squares regression [237,238] or deep learning [239] may offer alternative approaches to the use of spectral reflectance to assess plant physiological status through the integration of a wider multivariate range of reflectance characters including other proxies. This is outlined in more detail in Section 9 below.

## 7. LiDAR 

**Figure 7:**
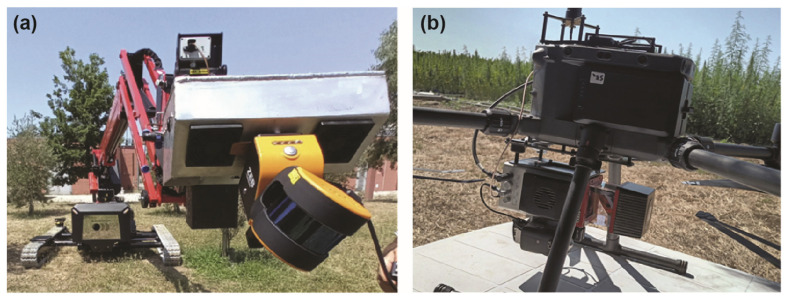
LiDAR systems mounted on: (**a**) a mobile phenotyping station (Geoslam Zeb Horizon), and; (**b**) an unmanned aerial vehicle (Riegel mini Vux).

Light detection and ranging (LiDAR) operates by emitting laser pulses (generally in the range of short infrared light: 830–1350 nm) toward a target, then measuring the time it takes for the light to bounce back and using these data to generate highly detailed 3D maps or models of the surroundings [240]. LiDAR can be operated on mobile, terrestrial, or aerial platforms, known as mobile laser scanning (MLS), terrestrial laser scanning (TLS), and airborne laser scanning (ALS), respectively (Figure 7). LiDAR appeared shortly after the invention of the laser in 1960 [241]. Airborne laser scanning with manned aircraft preceded terrestrial laser scanning, but swiftly gained ground because of its exceptional accuracy and versatility. The autonomous vehicles industry and topographic surveying predominantly dominate the demand for LiDAR technology. However, the demand also extended rapidly to other sectors such as urban planning, archaeology, environmental monitoring, forestry, and agriculture.

The latest technological advancement in sensor manufacturing led to a new generation of high-resolution, small-in-size, lightweight, and cost-effective LiDARs. At the same time, unmanned aerial vehicle (UAV) technology went through remarkable advancement in flight stability, autonomous capabilities, loading capacity, and battery life. Combining those two rapidly evolving technologies unleashed the race for unmanned aerial LiDAR for expedited, high-resolution, and cost-effective surveys. Today, both terrestrial laser scanning and airborne laser scanning offer precision and accessibility across various industries. As technology evolves and costs decrease, the demand for LiDAR for data collection and analysis will grow even further.

### 7.1. Application

An accurate and fast method for determining phenotyping traits is essential to select promising genotypes for crop breeding. The rapid, non-invasive, and high-resolution capabilities of LiDAR has revolutionized the field of phenotyping, enabling researchers to capture intricate information about plant height, canopy structure, leaf area, internode distance, and even the volume of individual plant organs, such as leaves and fruits [242]. These precise measurements enable scientists to gain deep insights into plant growth, stress responses, and overall health, contributing to advancements in crop breeding, precision agriculture, and ecosystem monitoring. Underwood et al. [243] used mobile laser scanning to map flower and fruit distributions to estimate and predict yield for individual almond trees. Estornell et al. [244] used airborne laser scanning to extract walnut structure parameters. They reported coefficient of determination values (R^2^) equal to 0.95, 0.87, and 0.83 for crown diameter, stem diameter, and stem volume, respectively. Li et al. [245] demonstrated the potential of airborne LiDAR in estimating canopy height, leaf area index, and biomass components of maize during the peak growing season. Sanz et al. [246] reported a strong correlation between tree row LiDAR volume and leaf area for apple, pear, and vine trees with R^2^ of 0.85, 0.84, and 0.86, respectively. Kang et al. [247] used LiDAR–camera fusion techniques to perform accurate fruit localization in the apple orchards. The reported standard deviations of fruit localization at 0.5, 1.2, and 1.8 m were 0.253, 0.230, and 0.285 cm, respectively. Tsoulias et al. [248] used two terrestrial laser scanning measuring tools at 660 and 905 nm to capture fruit number (R^2^ = 0.99), fruit size (R^2^ = 0.98), and chlorophyll content (R^2^ = 0.78) in the apple fruit skin as an indicator of ripeness. The authors in [249] developed an ensemble learning model for apple tree yield prediction using a combination of LiDAR and multispectral imagery from unmanned aerial vehicles (UAV). They found three features (crown volume, ratio vegetation index, and crown projection area) that contribute most to apple yield prediction. Wang et al. [250] used canopy features extracted from UAV LiDAR, hyperspectral, and thermal infrared sensors as a proxy to estimate sugar content in sugar beetroot.

### 7.2. Photogrammetry versus LiDAR

Digital photogrammetry and LiDAR are used to create 3D models to evaluate static and dynamic changes in structural and functional phenotypes. Photogrammetry generates a point cloud using alignment and overlapping techniques of digital optical images [251]. LiDAR instead reconstructs 3D models by measuring the rebounding light points emitted by the sensor itself (active sensor). Compared to photogrammetry, this characteristic makes the LiDAR unaffected by light conditions [252] or background reflectance [253]. In the case of aerial system, point cloud accuracy depends on the precision of the LIDAR (or the resolution of the camera) itself and the quality of the inertial navigation system (INS) (IMU and GNSS) system. Other important factors affecting accuracy are flight planning and execution (flight altitude, overlapping distance, and UAV speed), weather conditions (light, wind), and the techniques used in the pre- and post-processing. 

Another advantage of LiDAR is the large laser point density (it can easily exceed 200 points/m^2^) compared to the limited tie points generated from photogrammetry. Also, laser beams can infiltrate through dense canopy, reach the soil, and bounce back up, allowing direct measurement of the ground soil and the top canopy. Photogrammetry penetration capability is limited by the resolution of the camera and by the darkness and shadows created by the dense canopy. LiDAR can also record multiple returns from different layers of vegetation, thus providing accurate information related to canopy density, structure, and plant height.

## 8. Root Zone Phenotyping 

In addition to helping plants absorb water and nutrients from the soil, root apparatus also serves as a sensor of soil environmental stresses including heat, drought, and salt that influence plant adaptation to their environment [254]. Plants modify their root systems to maximize the availability of nutrients and water, which affects plant resilience and productivity [255]. An effective root system, adapted to respond effectively to soil and environmental changes, is crucial to maintain growth and yield under stress conditions [256,257,258]. Nevertheless, there are still several factors that need to be fully elucidated on how soil environmental stress conditions affect root morphology and architecture as well as root functional traits, such as nutrient and water uptake, root exudation, and interactions with soil microorganisms [259]. 

Root plasticity is the capacity of a given genotype to change phenotype under different environmental conditions [260], and is critical to the ability of a plant to withstand abiotic stresses. However, root plasticity is often overlooked by breeders due to the challenges associated with monitoring root development in soil [261]. Breeders can now take advantage of sophisticated systems and sensors to observe the growth of the root system and assess root uptake of water and nutrients from soil [262,263,264]. Genotype variation in root traits consists of several features, such as total biomass, root length, root angle, number of lateral roots, and nodal roots [254,261]. Additionally, resistance to soil pathogens/pests, nutrient/water uptake, and yield of root crops are agronomically important root traits that should be introduced into breeding programs. These breeding programs should also consider that root trait responses may vary between different environmental conditions [265]. 

Root phenotyping is important in terms of enhancing plant growth and quality ([264] and papers cited within). The emergence of plant phenomics has boosted the development of high-throughput phenotyping imaging technologies to study plant roots grown in soil [263,264,266]. The ability of plants to efficiently gather immobile and mobile soil nutrients, such as phosphate and nitrate, can be significantly impacted by architecture-scale features such as root branching and angle [267,268]. Moreover, anatomical traits such as root hair length and xylem size also provide abiotic stress tolerance [269,270]. Root system architecture (RSA), composed of structural features such as root length, spread, number, and length of lateral roots, is highly plastic in response to abiotic stress conditions that affect the growth and development of above-ground biomass [271]. Despite the importance of roots in supplying water and nutrients for photosynthesis and growth, and the critical role of root plasticity in helping plants to cope with abiotic stress [272], root traits have been often neglected by breeders because of the challenges associated with following root developing throughout an experiment [261]. Root angle in crops represents a key trait for the efficient capture of soil resources. A root angle regulatory gene called ENHANCED GRAVITROPISM1 (*EGT1*), encoding a putative anti-gravitropic component, has been recently characterized, with loss-of-function enhancing root gravitropism in cereals [273]. 

In spite of the challenges that plant roots intrinsically harbor for measurement through phenotyping, efforts to incorporate root traits into breeding programs have been mainly performed in cereals [254,274,275]. Analysis of roots through phenotyping is pivotal for the identification of root traits that are beneficial to crops, their integration into new cultivars during the pre-breeding process, and their management using precision agriculture [255]. Since soil is a biologically, chemically, and physically heterogeneous environment, monitoring the whole root system in a field is challenging. There are difficulties in identifying the ideal root systems for optimal crop growth due to variability in root development under diverse environmental conditions; nonetheless, several procedures and methods have been developed for root phenotyping. The increasing interest in root phenotyping is demonstrated by several reviews that have been published on this topic in recent years, considering both high-throughput phenotyping platforms and in the field [255,262,263,276,277]. Non-destructive techniques under controlled conditions are gaining interest due to the development of imaging and sensor technologies integrated within high-throughput root phenotyping platforms that enable RSA development to be tracked [262,263,278]. Conversely, the characterization of RSA in the field requires the application of laborious methods. Shovelomics, or root crown phenotyping, is one of the most widely adopted high-throughput methods to follow root architecture in plants grown in the field [279,280]. This protocol is based on the excavation of mature root crowns from the field followed by manual phenotyping, and was originally designed for maize before being applied to legumes and wheat [279,280,281]. Root crown phenotyping includes several steps: excavation, transport, soil removal, and measurement [281]. This process allows measurement of several parameters characterizing the upper portion of crop root systems, and may be combined with genome-wide association studies, QTL analysis, physiological measurements, and -omics techniques. More recently, novel sampling methods combined with digital imaging and novel software have been proposed for next-generation Shovelomics [282]. Mini-rhizotrons require the installation of a transparent tube into the soil, with a camera inserted to record root development [283,284]. This system allows the monitoring of root growth over time and tracing of single roots during their development [285]. The acquired images are analyzed using software that provides information on the root length and diameter. It is worth noting that this phenotyping system has been also used to follow the formation of root nodules in soybean [286]. Future developments in root system capacitance, ground-penetrating radar, and thermoacoustic imaging may enable non-invasive measurement of root architecture under field conditions [287].

### Phenotyping the Interaction of Roots and Soil Microorganisms 

Roots host soil microorganisms that can enhance plant growth and resource efficiency, as well as modulate the supply of resources, root-to-shoot signalling, plant growth, flowering, and productivity. These root-associated microorganisms play a fundamental role in plant adaptation to adverse environmental conditions [259]. Root traits influence the composition of root-associated microbes, which, in turn, can interact with the plant, subsequently modifying those root traits associated with the ability to explore and exploit resources present in the soil. Root features that influence the interactions with soil microorganisms, including beneficial ones, are architectural and morphological traits as well as the biochemical fingerprint profile the root exudates that contribute to the recruitment, colonization ability, and functional outline of the associated microbial communities [259,288]. How root architecture and exudates, which can change according to plant age, genotype, and environmental conditions, influence rhizosphere and root environments, and consequently the microbial recruitment, represents a hotspot in research into root–soil microorganism interactions. It is, therefore, important to phenotype roots for their capacity to interact with beneficial soil microorganisms, which positively affect nutrition, growth, stress tolerance, and disease resistance, with the aim of identifying crop genotypes that are able to take advantage of these interactions most effectively. 

## 9. Data Processing—Machine Learning Applied to Plant Phenotyping

Machine learning is a field of artificial intelligence that attempts to solve a learning problem from previously collected data. During the training phase, a prediction model is progressively tuned based on a part of the dataset while the other part of the dataset is usually kept to later assess the model performance (e.g., holdout method, cross-validation). In previous sections, we presented various physiological indicators and how to quantify these parameters. Machine learning can provide data-based indicators of plant status using high-throughput phenotyping data [289]. In particular, such indicators may gauge physiological values that would otherwise require expert observation or complicated ground truth measurements [196,290].

As for any data-driven model, the quantity, quality, and specificity of the data lead to different learning perspectives. We may distinguish raw input data in which part of the information needs to be extracted, such as RGB images, from more specific input data such as spectral reflectance indexes that provide a predefined abstraction of the data, which may be difficult to adapt further. In the meantime, the complexity of the model determines its capacity to learn relevant latent features and make up any required abstraction. Broadly speaking, a simple learning model requires more elaborate input data, whereas more elaborate learning models are more suitable to process raw data. 

After general considerations on the input data and their implications, we consider the principal machine learning methods that can be applied to plant phenotyping. We decided to separate the methods according to the complexity of the learning model between “shallow” and “deep” learning. This part does not pretend to provide an exhaustive discussion on machine learning methods, but rather present the most relevant approaches along with some insights on standard machine learning issues. 

### 9.1. Data Considerations

The feature learned by the model differ according to the input sensor and how the information is encoded. For instance, a rasterized image is more suitable to identify local spatial patterns, since it is the organization of the pixels rather than their designated values that is determinant. Therefore, such data are ideal for visual stress recognition [291], while LiDAR measurements provide the third dimension and help to implicitly discriminate the shrubs from trees in UAV sensing [292]. Though they are intuitively understood, the implications of the selection of data sources and how to combine these datasets is demonstrated only *a posteriori*, once the model has learned. Though studies in plant phenotyping have spontaneously learnt on several input sources, as in Ewald et al. [292], the reader may further explore the issues associated in extensive data fusion surveys [293], especially for complex learning models [294].

On top of the intrinsic information provided by the input data sources, all sensed data aggregate a great deal of other information that is linked to external factors that are not explicitly considered by the model, such as the weather, the growing stage of the plant, or the species. All these confounding variables restrict the features learned by the model for this specific context, and preclude the transfer of these learned features to other situations. An approach to overcome such situations is to make the model learn from wide and diverse datasets [295]; however, the complexity of the model should remain sufficiently accurate for each of these contexts.

### 9.2. Shallow Learning Approach

Linear regression is a straightforward method that proportionally maps a set of sensed variables with the target variable that the study wishes to retrieve. As the model is relatively simple, the input data only need to provide the relevant abstraction to linearly link to the target variable. As an example, using higher level information it is possible to linearly correlate spectral reflectance indices to physiological indicators of water stress in olive trees (Figure 6) [157]. As a first approach, this method may highlight immediate correlations between sets of variables, but it is sensitive to sample noise and the selection of the input variables.

Partial least square regression [296] is currently the main regression method employed in physiological experiments [223]. Partial least square regression is a composition between a principal component analysis and a linear regression. Prior to fitting the sampled variables on a line, the sampled variables are statistically recombined, so that the variance between samples is emphasized. This method is particularly suitable for multiple input variables that provide similar information, which is usually the case for hyperspectral reflectance (Figure 5). Thus, it has been possible to establish several strong correlations between reflectance and physiological values as *V*c_max_ *J*_max_, chlorophyll content, and nitrogen concentration for several species of plant from maize [297] to tropical trees [298]. For a more detail description please see the reviews of Gill et al. [299] and Grzybowski et al. [196].

The variables combined during partial least square regression represent a latent feature that acts as intermediary input data to a successive linear regression. An interesting feature of the partial least square regression approach relies on the capacity to interpret directly the feature learnt through the computed weight, also called ‘loading values’ in this case. These values may identify a direct link between the reflectance combination and the specific indicator of plant physiological status [297]. 

Other standard machine learning methods such as support vector machine, artificial neural networks, or random forest have, to a lesser degree, been employed for plant phenotyping, but mainly in the classification of plant biotic stress (for a more detailed review see [300]).

### 9.3. Deep Learning Approach

Deep learning is a machine learning technique that attempts to tackle the problem of the transferability of the features learned to different contexts such as crop species, climate, or agricultural treatments. As in partial least square regression, these models learn internal features but the number of layers is significantly larger. We may visualize a deep learning model as a stack of multiple layers of single machine learning models, where each succeeding layer uses the output from the previous layer as input. As the number of stacked layers increase, the features learned can then be generalized to more diverse contexts [301]. Such methods require a large amount of data, which make them more interesting from the perspective of elucidating physiological information from high-throughput phenotyping data.

In particular, a deep convolutional neural network (DCNN) is an appropriate method to deal with image data, since the connection between the layers is structured to extract spatially local features [301]. Hence, in plant phenotyping, deep convolutional neural networks have been applied to automatize recognition tasks that are difficult to solve with image processing solutions, but easy for a skilled human operator. The immediate application in plant phenotyping is to monitor crop productions by counting the fruits from UAV or terrestrial ground-based images [302] or plant stress [300]. In the context of plant genotype characterization, a deep convolutional neural network has been successfully inserted into the overall process to count the root tips from photographic images [303]. In plant physiology specifically, deep convolutional neural networks have been applied to non-destructively identify nutrient deficiency based on images of leaves [291] or assess plant water status using plant images combined with other sensors [304]. To the best of our knowledge, these methods have been mainly applied to RGB images, but interesting results could also be obtained considering spectral reflectance values as observed in the study of Rehman et al. [239]. 

The abstractions reached by deep learning models expand their applications over the class or value estimation. For instance, generative adversarial networks have also been used in plant phenotyping for data augmentation [305]. Instead of regressions, such models generate realistic images to increase the number of samples in the original dataset, and, therefore, enhance any successive machine learning process. Another promising perspective is to provide further insights on the latent features learned inside the deep learning model. The explainable AI framework applied to stress phenotyping allows the identification of the type of stress suffered by a leaf, but also the area of the leaf where this stress occurs [291]. This complementary approach may help us to understand the physiological process and build dedicated sensors to gauge plant physiological status for more effective phenotyping of traits associated with high productivity and climate resilience in crop varieties. 

## 10. Summary 

Plant physiological function forms the basis of productivity and resilience to abiotic stress. Traits such as the rate of photosynthesis, the capacity to dissipate excess energy, protective antioxidants, stomatal control, and the uptake of water/nutrients are central to plant growth rate, tolerance to abiotic stress, and water/nutrient usage. The characterization of physiological attributes associated with desirable traits is essential to successful plant phenotyping, to identify and then develop crop varieties with higher yields, lower input requirements (water, nutrients, pesticides etc.), and improved climate resilience. However, phenotyping efforts are constrained by the difficulties associated with accurate direct measurement of plant physiological processes, specifically those related to photosynthetic leaf gas exchange, ChlF, plant water relations, and below-ground processes. The use of low-cost optical sensors, remote/proximal sensing of spectral reflectance, SiF, and LiDAR offers the potential to rapidly assess plant physiological status at high temporal resolution over wide spatial scales. However, given the indirect nature of many of these measurements, empirical physiological data are still a necessity. Developments in leaf gas exchange technologies such as the integration of carbon isotopic analysis of CO_2_ may enable more rapid assessment of photosynthetic, photorespiratory, and metabolic respiratory fluxes of carbon. Future technological developments in LED and sensor technologies will improve the availability, expand the application, and reduce the costs of plant phenotyping and digital agriculture technologies. Partial least squares regression, deep-learning, and artificial intelligence technologies may enable the use of multi-variate data sources to accurately infer plant physiological performance. The rapidly developing nature of these technologies may alleviate the ‘physiological bottleneck’ that has constrained the application of phenotyping efforts to enhance future food security in a hotter drier world.
